# Spatial–Temporal Dynamics of Vegetation Indices in Response to Drought Across Two Traditional Olive Orchard Regions in the Iberian Peninsula

**DOI:** 10.3390/s25061894

**Published:** 2025-03-18

**Authors:** Nazaret Crespo, Luís Pádua, Paula Paredes, Francisco J. Rebollo, Francisco J. Moral, João A. Santos, Helder Fraga

**Affiliations:** 1 Centre for the Research and Technology of Agro-Environmental and Biological Sciences (CITAB), Institute for Innovation, Capacity Building, and Sustainability of Agri-Food Production (Inov4Agro), Universidade de Trás-os-Montes e Alto Douro (UTAD), 5000-801 Vila Real, Portugal; nazaret@utad.pt (N.C.); luispadua@utad.pt (L.P.); jsantos@utad.pt (J.A.S.); 2 Department of Agronomy, School of Agrarian and Veterinary Sciences, University of Trás-os-Montes e Alto Douro, 5000-801 Vila Real, Portugal; 3 School of Sciences and Technology, University of Trás-os-Montes e Alto Douro, 5000-801 Vila Real, Portugal; 4 LEAF—Linking Landscape, Environment, Agriculture and Food—Research Center, Instituto Superior de Agronomia, Universidade de Lisboa, Tapada da Ajuda, 1349-017 Lisboa, Portugal; pparedes@isa.ulisboa.pt; 5 Department of Graphic Expression, School of Agricultural Engineering, University of Extremadura (UEX), Avda. Adolfo Suárez, s/n., 06007 Badajoz, Spain; frebollo@unex.es; 6 Department of Graphic Expression, School of Industrial Engineering, University of Extremadura, Avda. de Elvas, s/n., 06006 Badajoz, Spain; fjmoral@unex.es

**Keywords:** olive orchards, MedPDSI, SAVI, NDMI, remote sensing

## Abstract

This study investigates the spatial–temporal dynamics of vegetation indices in olive orchards across two traditionally rainfed regions of the Iberian Peninsula, namely the “Trás-os-Montes” (TM) agrarian region in Portugal and the Badajoz (BA) province in Spain, in response to drought conditions. Using satellite-derived vegetation indices, derived from the Harmonized Landsat Sentinel-2 project (HLSL30), such as the Normalized Difference Moisture Index (NDMI) and Soil-Adjusted Vegetation Index (SAVI), this study evaluates the impact of drought periods on olive tree growing conditions. The Mediterranean Palmer Drought Severity Index (MedPDSI), specifically developed for olive trees, was selected to quantify drought severity, and impacts on vegetation dynamics were assessed throughout the study period (2015–2023). The analysis reveals significant differences between the regions, with BA experiencing more intense drought conditions, particularly during the warm season, compared to TM. Seasonal variability in vegetation dynamics is clearly linked to MedPDSI, with lagged responses stronger in the previous two-months. Both the SAVI and the NDMI show vegetation vigour declines during dry seasons, particularly in the years of 2017 and 2022. The findings reported in this study highlight the vulnerability of rainfed olive orchards in BA to long-term drought-induced stress, while TM appears to have slightly higher resilience. The study underscores the value of combining satellite-derived vegetation indices with drought indicators for the effective monitoring of olive groves and to improve water use management practices in response to climate change. These insights are crucial for developing adaptation measures that ensure the sustainability, resiliency, and productivity of rainfed olive orchards in the Iberian Peninsula, particularly under climate change scenarios.

## 1. Introduction

The olive tree (*Olea europaea* L.) is a perennial species known for its longevity and adaptability to the Mediterranean climate, where it has been cultivated for millennia [1,2]. Native to the Mediterranean basin [3], this species played a key role in the region’s cultures and economies, symbolizing Mediterranean agriculture [4,5]. Olive oil, the main product obtained, is essential in the Mediterranean diet, and it is extracted from olives using traditional or modern methods [6]. It is valued for its flavour, versatility, and nutritional properties [7]. Rich in monounsaturated fats, such as oleic acid, and antioxidants like vitamin E, olive oil offers various health benefits including cardiovascular protection and anti-inflammatory properties [8,9].

The main Iberian Peninsula (IP) countries, Spain and Portugal, are among the world’s leading global olive oil producers and play a key role in global supply chains, underscoring the regional major economic role of oliviculture [6,10,11]. During the period 2013 to 2023, olive oil production in Portugal experienced a significant annual increase of 6.9% (3.4 × 10^3^ t/year), while production in Spain recorded a negative trend of −4.9% per year during the same period (Figure S1). The IP presents both opportunities and challenges for olive cultivation [12]. The IP Mediterranean-type climate, which is typically characterized by hot dry summers and mild wet winters [13,14], is generally favourable for olive tree cultivation [15,16]. However, increasing concerns about excessive heat and prolonged droughts, exacerbated by climate change [17], pose a threat to rainfed olive orchards, potentially reducing yields and affecting olive oil quality [18]. While rainfed olive orchards are becoming less frequent and are being increasingly replaced by super-intensive irrigated orchard systems, some IP regions still maintain traditional plantation systems.

The “Trás-os-Montes” (TM) agrarian region in Portugal and the province of Badajoz (BA) in Extremadura, Spain, are renowned for their rich traditions in olive cultivation, being a key agrarian value chain for their regional economies. These regions are characterized by traditional rainfed plantations with low plant density (typically <100 olive trees/ha) [19,20], known for being well-adapted to local conditions and for the production of high-quality olive oil [15]. In the TM region, olive groves cover approximately 82 × 10^3^ ha. TM is the second-largest olive oil-producing region in Portugal, after Alentejo, accounting for about 15% of the total national olive oil production [21] with an increase in the last decade (Figure S1). The most representative varieties in TM include “galega portuguesa”, “cobrançosa de Trás-os-Montes”, “madural”, “verdeal de Trás-os-Montes”, “cordovil de Trás-os-Montes”, “picual”, “negrinha”, and “arbequina” [10,22].

The BA province is known for its extensive olive cultivation, with a total cultivated area of 237,504 hectares, of which 73% are rainfed olive plantations [23]. Similarly to TM, this province has experienced an increase in olive production over the past 10 years [23], supported by technological advances in systems management [24,25]. BA ranks as the third province in Spain for olive production, following Jaén and Córdoba [23]. The olive oil of this territory is characterized by its distinctive flavour, which comes from local varieties such as “arberquina”, “morisca de Extremadura”, “cornicabra”, “picual”, “verdial de Badajoz”, and “gordal sevillana” [26].

Drought and climate change are major concerns for olive production [27], as these environmental stresses can significantly disrupt the tree’s phenology cycle [28]. Although olive trees are naturally heat- and drought-tolerant, being well-adapted to the long Mediterranean hot and dry summers, they are not immune to the extreme conditions that are becoming more frequent with climate change [17]. Prolonged periods of drought and erratic weather patterns can severely affect phenology stages, such as flowering, fruit set, and ripening, resulting in lower yields and compromised fruit quality [18,29]. To maintain olive production under these challenging conditions, it is essential to implement water use management practices tailored to the phenological needs of the tree [17,30]. Ensuring adequate moisture during key growth periods is critical to maintaining both yield and quality [29].

For the efficient monitoring and management of olive plantations, applying suitable tools to assess the impacts of drought conditions and vegetation health is crucial [17,31,32]. Understanding how these factors may influence olive orchards is essential for effective management and production optimization [33]. Drought indicators and vegetation indices are usually employed to gain insights into water availability and overall impacts on the health of olive trees [3,34]. There are several climatic indices that can be used to identify drought conditions, such as the Standardized Precipitation Index (SPI) [35,36] at different temporal scales (e.g., 3, 9, and 12 months) and the Palmer Drought Severity Index (PDSI) [37]. Nonetheless, these well-known indices are not specifically targeted at olive trees and are more suited for annual crops. The MedPDSI (Mediterranean Palmer Drought Severity Index) is an adaptation of the PDSI, which provides a more precise measurement of drought severity for olive trees [28,38]. Furthermore, MedPDSI was chosen for this study over other drought indices, such as the SPI, as it demonstrates a more accurate representation of drought conditions over the Mediterranean [38] due to its ability to account for the unique hydrological and climatological characteristics of this region [39,40]. Moreover, its sensitivity to the different seasonal cycles of evapotranspiration and water availability for a deeper understanding of drought impacts on olive trees are both economically and culturally significant in Mediterranean countries [38], facilitating the development of effective mitigation strategies [41,42].

To assess the health and vigour of olive orchards, vegetation indices derived from data acquired by earth observation satellite systems offer continuous large-scale observations for agricultural management and environmental monitoring [43,44,45]. Vegetation indices such as the Soil-Adjusted Vegetation Index (SAVI) [46], Ratio Vegetation Index (RVI) [47], the Normalized Difference Vegetation Index (NDVI) [48], Crop Water Stress Index (CWSI) [49], and the Normalized Difference Moisture Index (NDMI) [50] can be applied for this purpose. In this study, the SAVI and NDMI were selected. On the one hand, the SAVI is adjusted for soil brightness effects in areas with sparse vegetation, provides a more accurate assessment of vegetation health, particularly in olive orchards where soil reflectance can distort readings [46], and assists in decision-making regarding irrigation [51] and fertilization [52]. On the other hand, the NDMI evaluates moisture content within vegetation, important for monitoring the water status of olive orchards [50]. By comparing near-infrared (NIR) and shortwave infrared reflectance (SWIR), the NDMI can be used to detect drought conditions and for effective water management, especially during water stress periods [53,54].

The objective of this study is to carry out a spatial–temporal analysis of the drought impacts on olive tree vegetation in the two IP olive-growing areas, TM and BA. To conduct this analysis, SAVI and NDMI data were used, exploring how climate variability, as indicated by the MedPDSI, influences rainfed olive groves, thereby gaining an understanding of the interactions between climate and vegetation in Mediterranean olive-growing regions from 2015 to 2023. Moreover, an assessment of the suitability of remotely sensed vegetation indices for the multi-temporal monitoring of such variations over time and for assessing the potential temporal relationships with MedPDSI was conducted.

## 2. Materials and Methods

### 2.1. Study Areas

**Figure 1:**
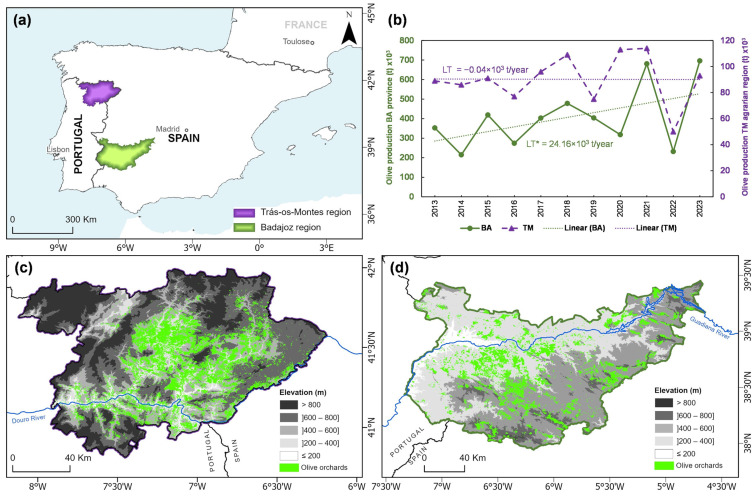
Study area and olive production in the territories of Badajoz province (Spain) and Trás-os-Montes agrarian region (Portugal). (**a**) Location and spatial extent of the study regions; (**b**) olive production (in tons) from 2013 to 2023 in both regions, along with the corresponding linear regression trends and linear trend values (LT); (**c**) elevation of TM (“Trás-os-Montes” agrarian region, Portugal) and olive orchard areas; and (**d**) elevation of BA (Badajoz province, Spain), olive growing areas. Data sources: INE (Statistics Portugal) and MAPA (Ministry of Agriculture, Fisheries and Food, Spain). * Significant at *p* < 0.05.

The study was conducted in two regions of the IP (Figure 1a), TM in northeastern Portugal (Figure 1c) and the BA province in the autonomous community of Extremadura, southwestern Spain (Figure 1d). The time series of olive yields in each region are shown in Figure 1b, along with the corresponding linear regression trends (linear trend values—LT—are also depicted).

#### 2.1.1. “Trás-os-Montes” Agrarian Region

Located in the northeast of mainland Portugal, TM is a mountainous region (−8.2° W, 40.5° N, −6° W, 42° N) covering an area of 12,283 km^2^. It borders with Spain to the north and east, forming part of the natural border between the two countries. The elevation in the region ranges from 70 m above sea level in the westernmost part of the Douro River valley to 1486 m in the extreme northeast (Montesinho Range).

**Figure 2:**
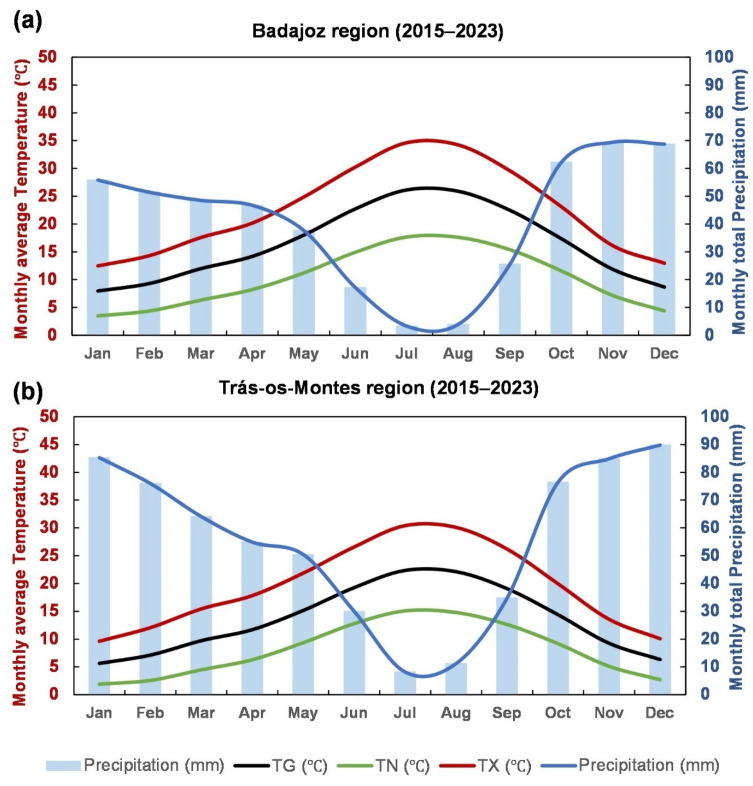
Ombrothermic diagrams for olive grove areas: (**a**) BA (Badajoz, Spain), (**b**) TM (“Trás-os-Montes” agrarian region, Portugal).

The climate of the TM region is classified as Mediterranean of type Csa according to the Köppen climate classification [13,55], characterized by warm, dry summers with maximum temperatures often exceeding 30 °C and mild, wet winters with precipitation concentrated between October and March (Figure 2). The region’s complex topography and varying altitudes (Figure 1c) contribute to the presence of a wide range of microclimates, where higher elevations commonly experience colder and moister winters and cooler summers compared to the lower elevation areas. The Douro River is the major water body flowing through the region, the second largest river in the IP, being important to the local agriculture and viticulture. Despite the region’s complex topography, it has developed well-adapted agricultural systems. Olive cultivation is of particular importance, with olive plantations covering a total area of 81,633 ha [21]. These small-scale olive groves are often located on terraced slopes, which helps preserve biodiversity and prevent soil erosion. The soils of the region are predominantly sallow, acid, and relatively poor in organic matter but well-drained, making them suitable for olive and grapevine cultivation [56]. The combination of traditional agricultural practices and local biodiversity makes this region an important area for the study of agroecological approaches within the IP. It is particularly renowned for producing very high-quality olive oil, with a distinctive flavour and unique organoleptic properties.

#### 2.1.2. Badajoz Province

The BA province, located in Extremadura, southwestern Spain, spans approximately 21,766 km^2^ (−7.5° W, 37.6° N, −4.5° W, 39.5° N) and is bordered by Portugal to the west. The region’s topography is predominantly flat, with elevations ranging from around 110 m in the lowlands near the Guadiana River to around 1110 m in the higher altitudes, such as the “Sierra de Tentudia” [57]. The Guadiana River is the main source of water for agricultural irrigation. The Guadiana basin is essential for sustaining agriculture in the region, making it a cornerstone of the local economy. The climate of the BA province is also classified as Mediterranean-type Csa according to the Köppen climate classification [13,55]. The soils in the BA province are predominantly characterized by a sandy loam texture, shallow depth, and low nutrient content [58]. In 2023, the region’s olive plantations covered approximately 237,504 ha [23]. These orchards are often integrated alongside the “dehesa” or “montado” agro-silvopastoral systems [59].

### 2.2. Data Collection

#### 2.2.1. Olive Orchard Data

The characterization of the olive orchard surface in the TM region was based on the digital inventory provided by the Portuguese Directorate-General for Territory (DGT) through the National Geographic Information System (SNIG), using the 2018 land-use and occupation charter of mainland Portugal (COS 2018). This product includes polygons representing different land-use and land cover classes, including olive orchards across TM within the permanent crops. For the BA region, the digital inventory was derived from the Copernicus Land Monitoring Service, using the CORINE Land Cover (CLC2018) dataset [60], with the Spanish responsible organization being the Autonomous Body National Centre for Geographic Information (IGN-CNIG), which, similarly to COS 2018, also includes polygon vector data indicating the land cover of olive groves within the permanent agriculture crops.

Both datasets were processed in ArcGIS Pro^®^ 3.2 (ESRI, Environmental Systems Research Institute) to extract and analyze the features of olive plantations and to ensure accurate delineations of these agricultural areas in the study areas, allowing for an analysis of the spatial distribution patterns of olive plantations in both TM and BA.

#### 2.2.2. Climate Data

Climate data for both regions were obtained from the E-OBS v29.0e gridded observational dataset, which provides an ensemble dataset on 0.1 regular grids [61]. E-OBS provides a comprehensive coverage of various meteorological variables across Europe, covering the whole of the IP. The dataset includes climate variables such as mean temperature (TG), daily minimum temperature (TN), daily maximum temperature (TX), daily precipitation (RR), and daily averaged sea level pressure (PP). While the E-OBS data range from 1 January 1950 to 31 December 2023, data covering only the study period were selected (from 2015 to 2023) in order to be comparable with the remote sensing data (c.f. next section). Although the dataset was originally provided with daily resolution, it was aggregated to a monthly resolution to facilitate the analysis of long-term trends and seasonal variations.

To calculate the MedPDSI, the model described in [40] was used, which required the use of TG, TN, TX, and RR data. For this model, given the data availability, reference crop evapotranspiration (ET_o_) was calculated using the Hargreaves–Samani method [62]. It is based on air temperature and is particularly useful in regions where data on solar radiation and humidity are scarce. This method has been shown to provides reliable estimates of ET_o_ [63], which is critical for accurately assessing drought conditions. Soil data, specifically the USDA soil classification scheme, are also required to compute the MedPDSI, which was obtained from the FAO Harmonized Global SOIL Database 2.0 [64]. These data were extracted into ArcGIS 3.2. using the implicit tool “Extract Values” for both regions. The classification of MedPDSI is based on the PDSI [37] and has been implemented and calibrated for mainland Portugal [65]. The index is computed using the modified version, resulting in a numerical value that indicates drought severity, typically ranging from −4 to +4, with negative values indicating drought and positive values indicating wetter-than-normal conditions (Table 1).

#### 2.2.3. Remote Sensing Satellite Data and Vegetation Indices

The remote sensing analysis included the calculation of vegetation indices, specifically the SAVI and NDMI, using harmonized multispectral data from the Harmonized Landsat Sentinel-2 (HLS) project, namely the HLSL30 product [66]. This seamless product is derived from the Operational Land Imager (OLI) sensor (onboard the Landsat 8 and 9 satellites) and the Multi-Spectral Instrument (MSI) sensor (onboard the Sentinel-2A and Sentinel-2B satellites). Harmonized data include “atmospheric correction, cloud and cloud-shadow masking, spatial co-registration and common gridding, illumination and view angle normalization, and spectral bandpass adjustment” [66].

The data were accessed and downloaded through the Google Earth Engine (GEE) platform, available from July 2015 to December 2023. This HLSL30 dataset provides consistent high-quality surface reflectance (SR) and top-of-atmosphere (TOA) data for vegetation analysis with a spatial resolution of 30 m. Together, these sensors enable global land observations every 2–3 days. Nevertheless, the GEE platform currently only provides the HLSL30 product, which includes data solely from the Landsat 8 and 9 satellites, with the above-mentioned corrections applied. Several spectral bands are available, such as coastal aerosol (B1), blue (B2), green (B3), red (B4), near-infrared (B5), two shortwave infrared bands (B6 and B7), cirrus (B9), and two-band TIRS (thermal infrared sensor) bands (B10 and B11). Additionally, it incorporates the quality bits (Fmask) band, which is used to identify cirrus clouds, cloud shadows, snow/ice, water, and aerosol levels.

The HLSL30 data availability is primarily determined by the acquisition schedule of these satellites, which is mostly limited by cloud cover. As such, to maintain consistent data quality, the dataset was filtered using the Fmask band to exclude pixels representing cirrus, clouds, cloud shadows, and those adjacent to clouds or shadows. A cloud-masking function is used to remove pixels affected by these conditions, ensuring that only clear-sky observations are included in the analysis. This step was crucial for minimizing noise due to atmospheric interference. The SAVI was calculated with the following equation [46]:

(1) $$SAVI=\frac{\left(NIR-RED\right)\times \left(1+L\right)}{NIR+RED+L},$$

where *NIR* represents the near-infrared band (B5), *RED* represents the red band (B4), and *L* is the soil adjustment factor, set to 0.5, to minimize the influence of soil brightness in areas with intermediate vegetation cover. SAVI values typically range from −1 to 1, with higher values indicating more vigorous vegetation (Table 2). The SAVI was computed for each acquisition available for the study period, and the maximum monthly SAVI pixel values were extracted for the extent of each study region and over the specified interval.

The NDMI was also calculated to assess vegetation moisture content, as in Equation (2) [54]:

(2) $$NDMI=\frac{NIR-SWIR}{NIR+SWIR},$$

where *NIR* is the near-infrared band (B5) and *SWIR* is the shortwave infrared band (B6). The NDMI is used to assess soil moisture content, with values generally ranging from −1 to 1, where higher values indicate higher moisture content in the vegetation (Table 3). The raster images were computed similarly to the SAVI, with the maximum monthly value derived for both areas and the study period.

The resulting SAVI and NDMI data were downloaded in raster format (TIF), resulting in 260 raster images (130 SAVI and 130 NDMI raster images) with a spatial resolution of 30 m. The raster images were loaded into ArcGIS Pro^®^ (version 3.2) along with the olive orchard cultivation polygons (Section 2.2.2). To extract SAVI and NDMI values within these polygons, the “Zonal Statistics as Table” tool in ArcGIS Pro^®^ 3.2 was used, allowing for the extraction and calculation of statistics of SAVI and NDMI values for each polygon representing olive cultivation areas, enabling an evaluation of its temporal changes.

To clarify the signal in the SAVI and NDMI, the Savitzky–Golay filter [69] was subsequently applied to smooth the data and improve the signal-to-noise ratio without distorting the signal. In this function, a degree of 5 was selected in order to minimize the fitting error and to minimize the sum of squares of the differences between the actual data points.

### 2.3. Data Analysis

To evaluate the relationships between MedPDSI values and vegetation indices (SAVI and NDMI), the non-parametric Spearman’s rank correlation coefficient was used at different monthly time lags. This method enabled the assessment of rank-based relationships between the MedPDSI and vegetation indices, inferring the time lag between drought occurrence and vegetation response. This approach helped to capture lagged correlations in the data not affected by outliers and ensured that both linear and nonlinear interactions were at least partially isolated. Moreover, monthly time series of MedPDSI, SAVI, and NDMI values were plotted for both regions, providing a visual representation of how drought severity fluctuated over time in the olive-growing areas of BA and TM and their association with vegetation health and moisture content. The monthly variations provide an understanding of the short-term dynamics on vegetation indices, capturing variations and seasonal patterns. By understanding these patterns and responses, it may become possible to infer the adaptive capacity and overall resilience of olive cultivation in the study areas.

Spatial analyses were also carried out using ArcGIS Pro^®^ 3.2 to visualize the distribution of the SAVI and NDMI across the olive-growing areas. Spatial data processing techniques, such as raster-based analysis, were used to create maps that illustrate the spatial gradients in these areas across the two regions. These maps may highlight areas with high and low values of the SAVI and NDMI, revealing the impact of drought conditions on olive plantations and enabling a comparison between BA and TM regions, namely in terms of drought vulnerability and vegetation response. This enables an analysis of the spatial heterogeneity within and between both regions. The results also allow for an assessment of local variations in drought severity and how these relate with the remote sensing indices.

The interpretation of the results requires a special focus on assessing how sensitive the vegetation indices employed in this study are to fluctuations in the MedPDSI and how well they capture the effects of drought conditions on olive orchards. Moreover, to further validate the accuracy and reliability of the results obtained, they are also compared with additional data sources of historical records of olive production and climate data from the Portuguese National Statistics Institute [21] or the Spanish Ministry of Agriculture, Fisheries, and Food [23].

## 3. Results

### 3.1. Climate and Vegetation Data

The analysis of the land cover datasets revealed that olive plantations at BA are located at lower elevations, with 92% of the olive groves located below 600 m compared to TM, where only 69% of olive groves are found below 600 m (Figure S2). Furthermore, olive orchards in TM are located in steeper areas than in BA. The average altitude in TM is 510 m, while in BA, it is 399 m.

Regarding the climatic conditions in the olive orchard areas, the analysis of the ombrothermic diagrams for the BA and TM regions in the period of 2015–2023 show some noteworthy differences (Figure 2). In BA, the climate mean (averaged over the above-mentioned historical period) monthly temperatures fluctuate between 7 and 27 °C. The summer maximum temperatures exceed 34 °C, with peaks typically occurring in July and August. Winters are relatively mild, with climate mean average temperatures ranging from 7 to 11 °C. In contrast, TM has monthly mean temperatures ranging from 5 to 23 °C and experiences climate mean maximum summer temperatures exceeding 30 °C, though not as high as those in BA. Winter temperatures in TM are also cooler than in BA, with average minima between 2 and 6 °C. These differences are much more accentuated when considering the two regions as a whole and not the olive orchard land cover areas, thereby suggesting that the most suitable areas for oliviculture have been selected by farmers throughout multiple generations.

The annual precipitation totals in olive grove areas were 400–500 mm in BA and 600–700 mm in TM. In BA, from October to April, monthly precipitation ranges from 50 to 70 mm, while in TM, it ranges from 70 to 90 mm. Both regions follow a similar Mediterranean-type seasonal precipitation pattern, with precipitation peaking in autumn, winter, and early spring (winter half of the year). Conversely, in summer, precipitation levels decline sharply, particularly in BA, where the dry season is more intense, with negligible precipitation from June to September. This indicates a more pronounced and longer dry period in BA, whereas TM, although also experiencing reduced summer rainfall, maintains slightly higher precipitation levels than BA. The higher latitude of TM and the corresponding stronger exposure to Atlantic air masses and westerly low-pressure systems explain this difference.

### 3.2. Droughts and Vegetation Indices

#### 3.2.1. Drought Spatial and Temporal Assessment

**Figure 3:**
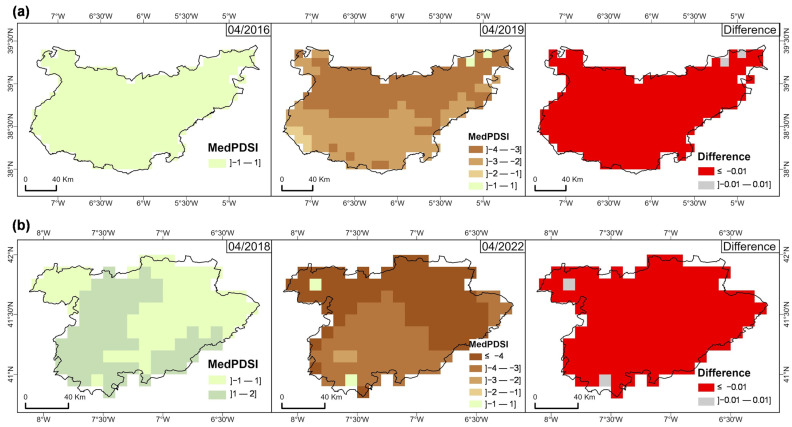
MedPDSI value index for olive grove areas: (**a**) BA (Badajoz province, Spain), (**b**) TM (“Trás-os-Montes” agrarian region, Portugal).

Figure 3 illustrates the spatial and temporal variations in MedPDSI across wet and dry periods observed in the BA and TM regions. A shift from normal conditions in April 2016 to severe drought conditions in April 2019 is observed in the BA province (Figure 3a), with negative values indicating an increase in drought intensity. Likewise, severe drought conditions are observed in the TM agrarian region (Figure 3b) in April 2022 in contrast to April 2018, especially in the northern and western parts.

In the BA province, the MedPDSI map for April 2016 shows predominantly positive values between 1 and −1, indicating normal-to-wetter-than-normal conditions across the area and favourable climatic conditions, with sufficient rainfall and no significant drought stress. However, in April 2019, a different scenario is observed, with the northern half of the region having lower MedPDSI values, particularly in the ranges of −2 to −3 (moderate drought) and −3 to −4 (severe drought). This shift was driven by a sharp decline in precipitation and a subsequent increase in drought severity over this three-year period. The difference map comparing 2019 and 2016 indicates widespread drought intensification in the BA province.

For TM (Figure 3b), the MedPDSI map for April 2018 reflects relatively balanced conditions, ranging from normal conditions in the eastern and southwestern areas to slightly humid conditions in the northwest. By April 2022, however, a significant expansion of extreme drought conditions (MedPDSI values ≤ −4) is observed in the northern and western areas of the region, with severe drought (MedPDSI values between −3 and −4) in the central and eastern parts. The difference map between April 2022 and April 2018 shows a general decline in MedPDSI values, indicating a substantial decrease in precipitation and an overall intensification of drought severity over the four years, with impacts of prolonged dry conditions, mostly in the central and eastern parts of TM, where the drought was more intense. A comparison of the MedPDSI between the two regions is shown in Supplementary Figure S3.

#### 3.2.2. SAVI Analysis

**Figure 4:**
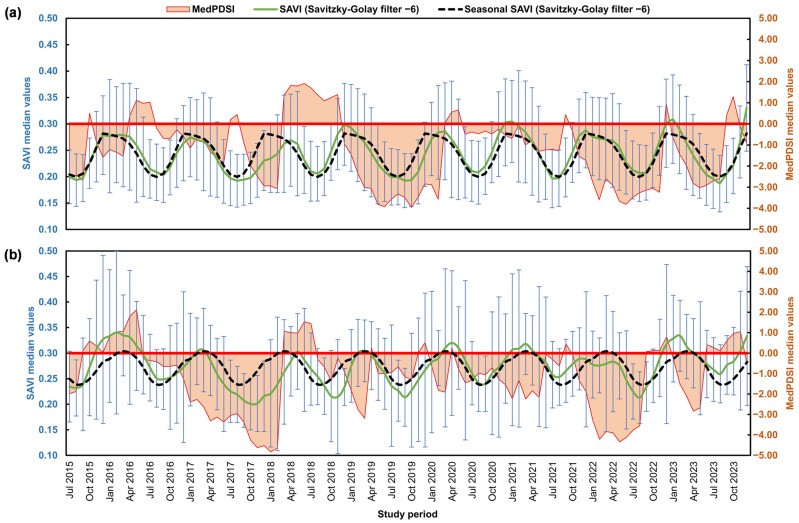
Comparison of median Soil-Adjusted Vegetation Index (SAVI) values (Savitzky–Golay filter) and Mediterranean Palmer Drought Severity Index (MedPDSI) values in (**a**) BA (Badajoz province, Spain) and (**b**) TM (“Trás-os-Montes” agrarian region, Portugal).

In the BA region (Figure 4a), the MedPDSI reached its lowest value in October 2019 (−3.96), reflecting a severe drought period, while the highest value occurred in June 2018 (1.91), reflecting a wetter period. The SAVI analysis shows that vegetation values generally decreased during drought periods, with the lowest value observed in August 2023 (0.19). In December 2023, the SAVI reached its highest value (0.33), which coincided with more favourable conditions for vegetation growth. The seasonal fluctuation of the SAVI reflects the expected annual vegetation cycle, with a peak around spring and a decline thereafter as a clear footprint of the dry season on vegetation vigour.

For TM (Figure 4b), the lowest MedPDSI value was recorded in January 2018 (−4.83), suggesting a severe drought, with the highest value in May 2016 (2.13) corresponding to a wetter period. The SAVI in TM reached its highest value in February 2016 (0.34) and its lowest in October 2017 (0.20), reflecting the vegetation’s response to more severe climatic conditions. The seasonal variation in SAVI shows higher values in early spring (March, 0.30) and lower values in late summer (September, 0.24) when the effects of drought and heat were more intense. The driest periods occurred between June 2016 and February 2018, with a severe drought in 2017, and from October 2021 to September 2022.

**Figure 5:**
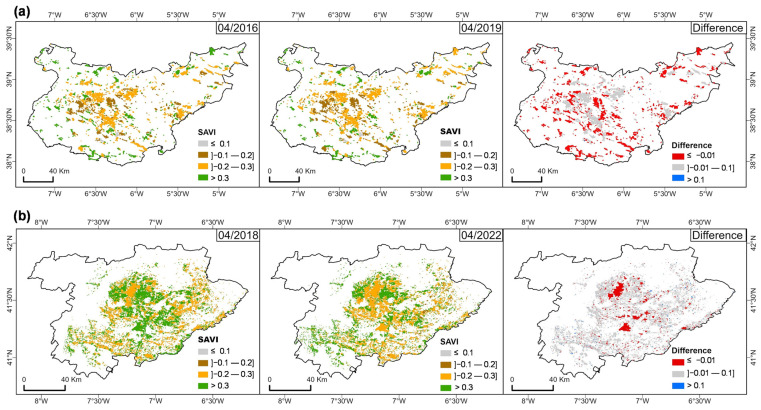
Soil-Adjusted Vegetation Index (SAVI) median values for olive orchard areas in (**a**) BA (Badajoz province, Spain) and (**b**) TM (“Trás-os-Montes” agrarian region, Portugal).

Figure 5 shows the spatial and temporal variations in the median SAVI values for the study regions when comparing two different periods, namely April 2016 with April 2019 in BA (Figure 5a) and April 2018 with April 2022 in TM (Figure 5b). Each comparison includes a map highlighting the differences in the SAVI values of the olive-growing polygons between wet and dry months.

For BA (Figure 5a), April 2016 represents a wet month, with most of the area displaying SAVI values indicating sparse vegetation cover (low vegetative development) or water stress conditions (Table 2, 0.1–0.3), with some healthier vegetation patches (>0.3), particularly in the southeastern part. By April 2019, a drier month, similar conditions prevailed, though more olive-growing areas presented values in the ranges of 0.1–0.2 and 0.2–0.3, indicating a decline in vegetation health. The difference map (2019–2016) shows a decrease in SAVI values (≤−0.01), especially in the central and eastern areas, suggesting increased vegetation stress or degradation during this period.

For TM (Figure 5b), April 2018 represents a wet month where the majority of the area had SAVI values in the 0.2–0.3 range, indicating potential water stress, with patches of healthier vegetation (>0.3) mainly concentrated in the central part of the region. By April 2022, a drier month, the SAVI distribution remained generally similar. However, there was a slight expansion in areas with lower SAVI values (0.1–0.2), indicating increased stress in olive orchards, and a shift in healthier zones, where SAVI values decreased from >0.3 to the 0.2–0.3 range. This pattern points to a general decline in vegetation health across the region. The difference map comparing these two periods shows a decrease in SAVI values (≤−0.01), especially in the central area, suggesting increased stress or degradation. In both maps of differences, there are areas where vegetation shows no significant changes in SAVI values between the compared periods (marked in grey), suggesting that vegetation health and cover remained relatively stable in these grey areas.

#### 3.2.3. NDMI Analysis

**Figure 6:**
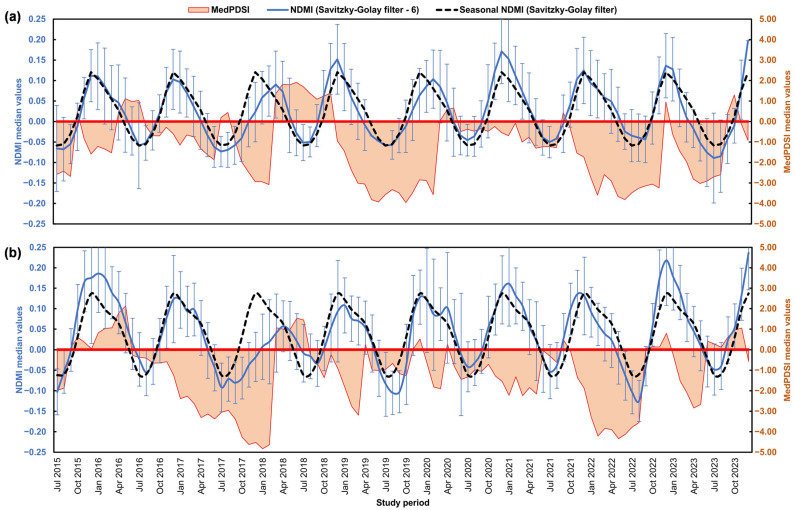
Comparison of median Normalized Difference Moisture Index (NDMI) values (Savitzky–Golay filter) and Mediterranean Palmer Drought Severity Index (MedPDSI) values in (**a**) BA (Badajoz province, Spain) and (**b**) TM (“Trás-os-Montes” agrarian region, Portugal).

The comparison of the MedPDSI and the NDMI is presented in Figure 6 for both study regions. In the BA region (Figure 6a), the NDMI shows a peak value of 0.20 in December 2023. The lowest value of −0.09 occurred in July 2023, suggesting significant water stress. Generally, the NDMI fluctuated between −0.09 and 0.20, with most months showing values below 0.20 in December 2023, indicating moderate water stress during most of the year. The seasonal NDMI (Savitzky–Golay filter) fluctuates between −0.06 in July and 0.12 in December, corresponding to the dry summer period and increased water stress and to the end of the growing season, respectively.

In the TM region (Figure 6b), the highest NDMI value was also recorded in December 2023 (0.24), while the lowest value occurred in August 2022 (−0.125), indicating that this region experiences similar seasonal trends in vegetation water content. The seasonal NDMI values range from −0.062 in July to 0.14 in December, reflecting the expected seasonal cycle of water content in vegetation and its alignment with climatic conditions.

The two driest periods in TM correspond to June 2016 to February 2018, with the most severe drought in 2017, and from October 2021 to September 2022. During these prolonged drought periods, the NDMI values were impacted, particularly in 2017 and 2022, where water content in vegetation was likely being affected due to sustained water stress. For both regions, NDMI values become critical when they drop below 0, especially during periods of negative MedPDSI, which indicates drought conditions.

**Figure 7:**
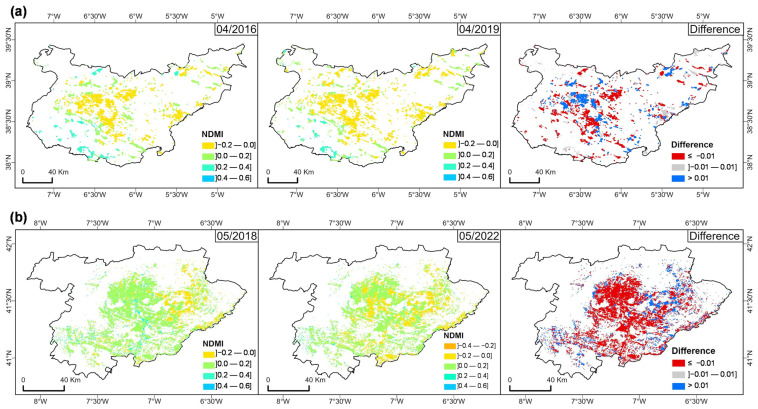
Normalized Difference Moisture Index (NDMI) median values for olive grove areas in March 2016 and 2019 in Badajoz (BA) province; (**a**) in May 2018 and 2022 in “Trás-os-Montes” (TM) agrarian region; (**b**) and the difference between the two periods in each region.

The analysis of NDMI variations over time reveals important soil moisture dynamics in the olive orchard areas of BA (Spain) and TM (Portugal), as presented in the comparison shown in Figure 7. For BA (Figure 7a), April 2016, a relatively wet month, can be compared to April 2019, a drier month. The NDMI maps show a reduction in moisture availability across most of the olive areas in the region. In both months, lower NDMI values (−0.2–0.0) were predominant, particularly in the central and eastern areas, suggesting areas of either mid-to-low canopy cover with high water stress or low canopy cover with moderate water stress. Similarly, for TM (Figure 7b), the drying pattern intensifies when comparing a wet month (May 2018) to a drier month (May 2022). The NDMI maps reveal a shift toward lower NDMI classes, from a predominant range of (0.0–0.2) in the wetter period to a range of (−0.2–0) in the drier period, suggesting increased water stress across olive orchards.

The difference maps for both regions, illustrating changes in the NDMI between the two periods (Figure 7), show that the difference map predominantly shows negative NDMI changes for the BA region (Figure 7a), especially in central, southwestern, and eastern areas, indicating a reduction in moisture levels. However, positive NDMI values are also apparent in some parts, suggesting that water availability in this region may directly depend on irrigation. Similarly, the TM region’s difference map displays widespread negative NDMI changes (Figure 7b), pointing to a generalized drying trend, likely due to prolonged drought conditions impacting natural water availability. A comparison of the SAVI and NDMI between the two regions is shown in Supplementary Figure S4.

### 3.3. Regional Comparisons

**Figure 8:**
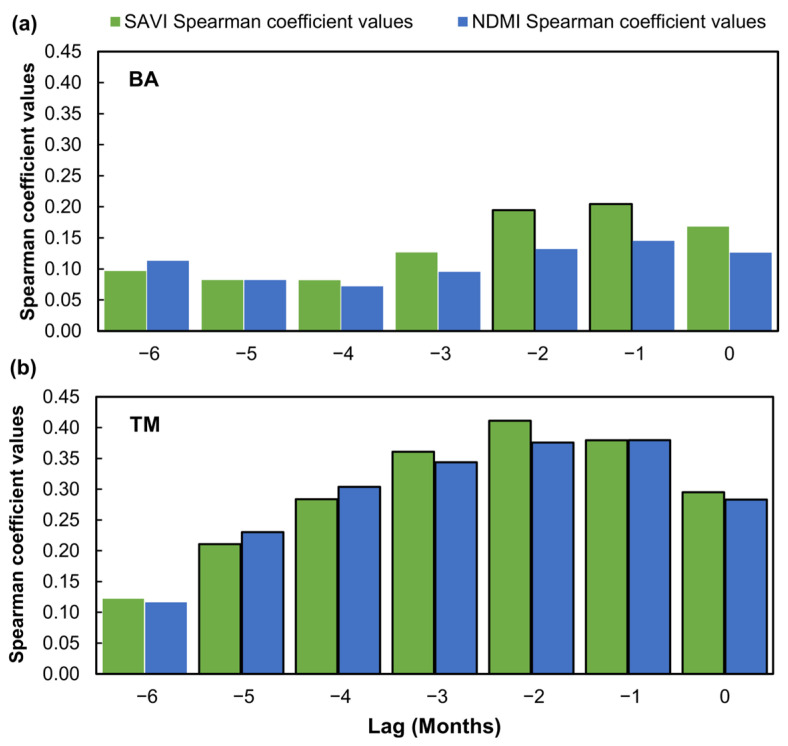
Auto-correlograms of Spearman coefficient values for the SAVI and Normalized Difference Moisture Index (NDMI): (**a**) BA (Badajoz, Spain), (**b**) TM (“Trás-os-Montes” agrarian region, Portugal). Bars with black contours are significant at *p* < 0.05.

The auto-correlograms using the Spearman coefficient values for both the BA and TM regions reveal distinct temporal autocorrelation patterns for the SAVI and NDMI indices. In the BA region (Figure 8a), only the SAVI shows a slight increase in autocorrelation at a lag of two months prior, suggesting an influence of past drought conditions on vegetation health response. This confirms that the structural characteristics of vegetation, as measured by the SAVI, may respond to environmental conditions with a delay, reflecting a certain level of resilience or temporal dependency. On the other hand, the NDMI in the BA region shows no significant autocorrelation at any lag, suggesting that this index is more immediately responsive to current conditions rather than influenced by previous climatic conditions.

The TM region (Figure 8b) shows a more consistent autocorrelation pattern, with both the SAVI and NDMI showing increasing Spearman coefficients from lag −6 to lag −2, followed by a slight decrease. This suggests a more constant temporal response in the TM region, where vegetation conditions have a delayed but stable reaction to past environmental conditions.

## 4. Discussion

The spatial–temporal dynamics of vegetation indices from remote sensing satellite data in olive orchards are addressed across two distinct regions of the IP, TM in Portugal and BA in Spain (Figure 1). The responses to drought conditions over almost eight years are evaluated. This research is important given the crucial role played by olive orchards in the socio-economy and culture of the IP, as Spain and Portugal are among the world leaders in olive oil production (Figure S1). The study’s main findings highlight the impacts of drought-based disturbances on olive orchards in both BA and TM. The climatic characteristics of these regions reflect the differences in growth conditions, such as latitude, orography, and elevation, which determine meso and microclimates that determine the geographical distribution and olive orchards. BA, with its lower elevation orchards [15], is subjected to more intense drought conditions, particularly during the summer months, compared to TM, which has slightly higher precipitation levels along with a more balanced seasonal distribution of rainfall (Figure 2).

The differences in the corresponding ombrothermic diagrams (Figure 2) should be considered, as according to Martins et al. [70], climate variations impact vegetation growth and agricultural productivity. The low summer rainfall is more noticeable in BA, where the dry season can last, on average, for five or more months, suggesting that olive groves in BA face greater water stress, potentially affecting vegetation growth and yield more severely than in TM. Similar patterns of seasonal precipitation decline have been observed across other Mediterranean regions, such as in southern Spain [71].

MedPDSI has shown to be a valuable tool in assessing drought impacts on olive orchards, revealing distinct drought periods in both regions (Figure 3). In the case of BA, severe droughts occurred in 2016, 2019, and 2022, while TM experienced intensified droughts from 2017 to 2018 and 2022, especially in the northern and western parts. According to Wang et al. [72], 59% of olive orchards were affected by the 2022 drought, coinciding with the driest year in both regions, causing significant impacts on agricultural productivity during that period. These results are consistent with those of Ezzine et al. [73], who observed similar drought impacts on vegetation in North Africa comparable with the NDMI.

The analysis of the SAVI (Figure 4) and NDMI (Figure 6) time series reinforces the seasonal variability in vegetation health observed in both regions. In BA, higher SAVI values are registered in December, while in TM, the SAVI peaks during spring and the NDMI during winter for both regions, coinciding with the wettest months. These patterns align with seasonal precipitation and phenological cycles, where winter precipitation promotes vegetation recovery and soil moisture and dry summers lead to water stress. This seasonal trend was also observed by Ozelkan et al. [74], who analyzed the relationship between drought indices (SPI) and vegetation indices (Normalized Difference Vegetation Index) in Turkey. The integration of vegetation indices with the MedPDSI offers a robust basis for adaptive water resource management and agricultural planning [39]. The NDMI is important for assessing water availability in vegetation, and together with the SAVI, these indices provide a comprehensive view of olive orchards particularly suited to the Mediterranean environment [75,76]. For instance, the NDMI can also indicate areas of irrigation or soils with high water retention capacity, even during summer, as observed in the Tierra de Barros area, the central region of BA (Figure 1d). Furthermore, the relationship between SAVI values above 0.3 and low or negative MedPDSI levels underscores its importance in monitoring drought stress and vegetation vigour in olive groves. This threshold highlights how the SAVI effectively reflects vegetation health, particularly under challenging Mediterranean conditions like those in BA and TM. These insights demonstrate the value of combining the SAVI and NDMI to understand the impacts of seasonal variability and water availability on olive orchards, providing actionable data for managing drought stress and maintaining agricultural productivity.

The spatial analysis of vegetation stress through SAVI difference maps for wet and dry months in both regions further confirms the impact of drought on vegetation health. The BA region appears more vulnerable to drought-induced vegetation stress, likely due to its lower elevation and more pronounced dry period, which could lead to a greater risk of long-term degradation (Figure 5). In contrast, TM shows slightly higher resilience, though prolonged droughts can still affect olive groves (Figure 7). This differential response to drought stress between regions aligns with the results of Bchir and Masmoudi-Charfi [77], who observed similar patterns in olive orchards across different Mediterranean microclimates.

The rank-based correlation analyses between the MedPDSI, SAVI, and NDMI highlighted their responsiveness to drought conditions (Figure 8). Both indices were found to be reliable indicators for assessing drought impacts, with the NDMI being effective at reflecting moisture stress for this crop [75]. Temporal autocorrelation patterns revealed a delayed but stable response of vegetation to past environmental conditions, suggesting resilience or temporal dependency. This was more evident in the TM region (Figure 8b), where a lag of −2 months was found to be particularly useful in indicating a longer-term reaction to drought stress. In contrast, the absence of significant temporal dependency for the NDMI in BA might be attributed to greater climatic variability or specific soil moisture dynamics. The higher autocorrelation values observed at negative lags in the TM region suggest that vegetation retains the influence of prior conditions, likely due to environmental factors such as soil characteristics or moisture retention capacity. These factors may buffer vegetation against short-term fluctuations, enabling a more stable response to prolonged drought stress. The observed lagged vegetation responses highlight the potential for these indices to serve as early warning indicators for drought conditions. These lagged responses can be incorporated into drought monitoring systems, allowing for the development of more proactive and adaptive drought risk management strategies, such as adjusting irrigation schedules and variety selection, mitigating the impact of drought on olive productivity.

However, the study’s relatively short time frame of eight years may limit its ability to capture long-term climate trends, which is related to the remote sensing dataset. This duration may not be sufficient to observe the full range of climate variability and its cumulative impact on olive cultivation. Future research could extend the temporal scope and include additional variables, such as soil characteristics and topography, to better understand drought impacts on olive cultivation while also focusing on improving these tools to better differentiate between rainfed and irrigated areas. This distinction is essential for drought monitoring and water use management, as it would enable better interventions and resource allocation. By addressing these aspects, future studies can provide a deeper understanding of the resilience and adaptability of olive trees to climate change, contributing to more sustainable olive grove management practices. Nevertheless, the results align with research from other major olive-producing regions. For instance, studies in Spain [75] and Chile [78] report similar challenges due to drought stress in olive orchards, highlighting the need for regional adaptation strategies.

## 5. Conclusions

This study highlights the urgent need for adaptation strategies for olive production across the IP, particularly in response to intensified droughts and climate change. In rainfed plantations, these challenges call for adequate irrigation strategies to optimize water use during critical drought periods, duly considering the sustainability of already scarce water resources. Satellite-based vegetation indices, such as the SAVI and NDMI, can be used as a tool for the large-scale monitoring of olive groves. While the SAVI is useful for assessing vegetation health in areas with sparse plant coverage, the NDMI can be used for the identification of areas with higher water retention, such as irrigated zones. This ability enables more accurate, region-specific water management, improving drought resilience and supporting economically and environmentally sustainable olive production.

The integration of the SAVI, NDMI, and the MedPDSI provides a robust framework for monitoring water stress during drought periods and guiding agricultural planning. Satellite-based monitoring circumvents geographic and regulatory challenges, offering consistent, cost-effective data collection that may feed decision support in a precision agriculture context. With advances in satellite technology, drought-resistant cultivars, and smart irrigation practices, the olive industry can adapt to climate change while maintaining sustainability and productivity, also contributing to the world’s food safety and security.

## Figures and Tables

**Table 1:** Range values for MedPDSI and its classification. The classification of MedPDSI is based on the PDSI [37] and has been implemented and calibrated for mainland Portugal [38].

Values	MedPDSI Classes	Colour Code
≥4	Very humid	
3–4	Severe humid	
2–3	Moderate humid	
1–2	Slightly humid	
−1–1	Normal conditions	
−2–−1	Drought-neutral conditions	
−3–−2	Moderate drought	
−4–−3	Severe drought	
≤−4	Extreme drought	

**Table 2:** Range values for SAVI and interpretation. SAVI classification based on NDVI values, adapted from Quille-Mamani et al. [67].

Values	Interpretation	Colour Code
<0.1	Bare ground, water bodies, clouds	
0.1–0.2	Sparse vegetation cover or areas	
0.2–0.3	Water stress	
>0.3	Healthy and dense vegetation	

**Table 3:** Range values for NDMI and interpretation. NDMI classification based on Antognelli [68].

Value	Interpretation	Colour Code
≤−0.8	Bare soil	
−0.8–−0.6	Almost absent canopy cover	
−0.6–−0.4	Very low canopy cover	
−0.4–−0.2	Low canopy cover, dry or very low canopy cover, wet	
−0.2–0	Mid-low canopy cover, high water stress or low canopy cover, low water stress	
0–0.2	Average canopy cover, high water stress or mid–low canopy cover, low water stress	
0.2–0.4	Mid–high canopy cover, high water stress or average canopy cover, low water stress	
0.4–0.6	High canopy cover, no water stress	
0.6–0.8	Very high canopy cover, no water stress	
>0.8	Total canopy cover, no water stress/waterlogging	

## Data Availability

All data used to elaborate this study are publicly available.

## Supplementary Materials

The following supporting information can be downloaded at: https://www.mdpi.com/article/10.3390/s25061894/s1, Figure S1. Annual olive oil production in Spain (left *y*-axis) and Portugal (right *y*-axis) from 2013 to 2023. Data sources: Statistics Portugal (INE, 2024) for Portugal and Ministerio de Agricultura, Pesca y Alimentación (MAPA, 2024) for Spain. * Significant at *p* < 0.05; Figure S2. Histogram of elevation in both regions: (**a**) Badajoz (BA) region; (**b**) “Trás-os-Montes” (TM) agrarian region; Figure S3. Comparison of MedPDSI between Badajoz (BA) and “Trás-os-Montes” (TM) agrarian region.; Figure S4. Comparison of SAVI and NDMI: (**a**) Badajoz (BA) region, (**b**) “Trás-os-Montes” (TM) agrarian region.
