# Supplementary material for: Spatial–Temporal Dynamics of Vegetation Indices in Response to Drought Across Two Traditional Olive Orchard Regions in the Iberian Peninsula
Source: Sensors (Basel). 2025 Mar 18;25(6):1894. doi: 10.3390/s25061894 (PMC11946650; doi:10.3390/s25061894)
Supplement: Supplementary file 1 [file sensors-25-01894-s001.zip › sensors-3450396-supplementary.pdf]

sensors

MDPI

Spatial-temporal Dynamics of Vegetation Indices in Response to Drought across Two Traditional Olive Orchard Regions in The Iberian Peninsula

Nazaret Crespo [1,2,3], Luís Pádua [1,2,4], Paula Paredes [5], Francisco J. Rebollo [6], Francisco J. Moral [7], João A. Santos [1,2,4] and Helder Fraga [1,2,3,*]

1 Centre for the Research and Technology of Agro-Environmental and Biological Sciences (CITAB), University of Trás-os-Montes e Alto Douro (UTAD), 5000-801 Vila Real, Portugal; nazaret@utad.pt, luispadua@utad.pt, jsantos@utad.pt, hfraga@utad.pt
2 Institute for Innovation, Capacity Building and Sustainability of Agri-Food Production (Inov4Agro), University of Trás-os-Montes e Alto Douro (UTAD), 5000-801 Vila Real, Portugal; nazaret@utad.pt, luispadua@utad.pt, jsantos@utad.pt, hfraga@utad.pt
3 Department of Agronomy, School of Agrarian and Veterinary Sciences, University of Trás-os-Montes e Alto Douro, 5000-801 Vila Real, Portugal; nazaret@utad.pt, hfraga@utad.pt
4 School of Sciences and Technology, University of Trás-os-Montes e Alto Douro, 5000-801 Vila Real, Portugal
5 LEAF—Linking Landscape, Environment, Agriculture and Food—Research Center, Instituto Superior de Agronomia, Universidade de Lisboa, Tapada da Ajuda, 1349-017 Lisboa, Portugal; pparedes@isa.ulisboa.pt
6 Department of Graphic Expression, School of Agricultural Engineering, University of Extremadura (UEX), Avda. Adolfo Suárez, s/n., 06007 Badajoz, Spain; frebollo@unex.es
7 Department of Graphic Expression, School of Industrial Engineering, University of Extremadura, Avda. de Elvas, s/n., 06006 Badajoz, Spain; fjmoral@unex.es

* Correspondence: hfraga@utad.pt

SUPPLEMENTARY MATERIAL

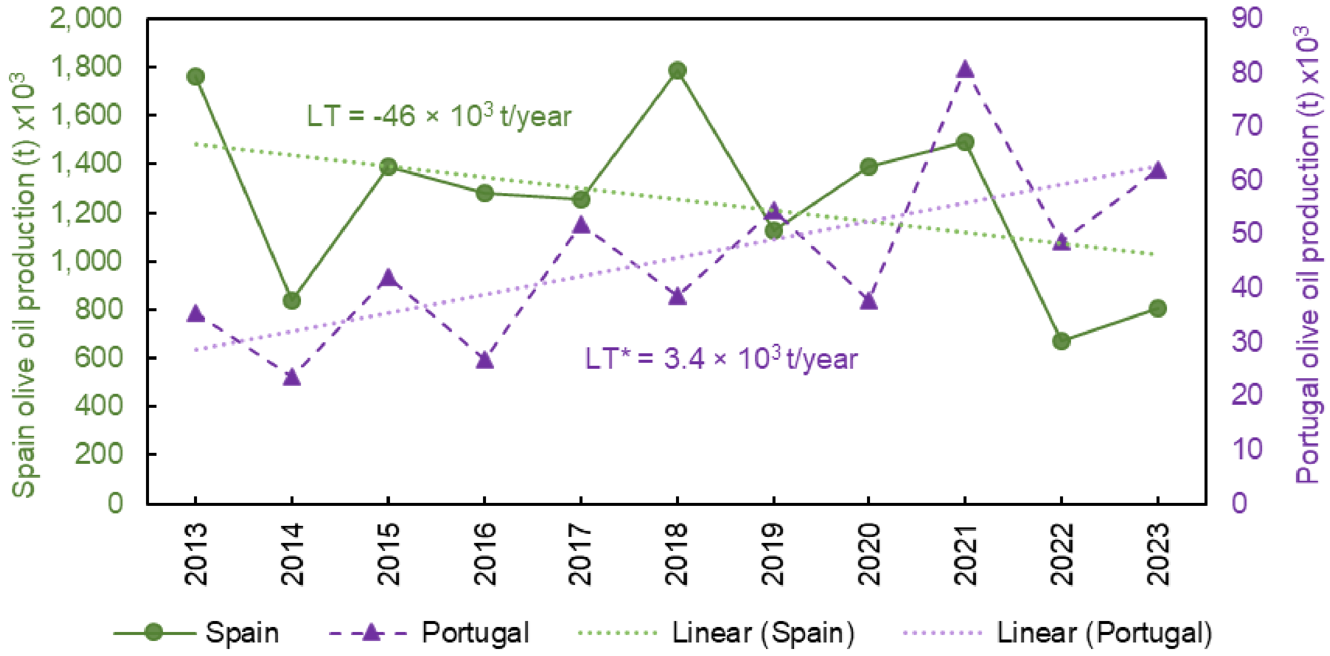

Figure S1. Annual olive oil-production in Spain (left y-axis) and Portugal (right y-axis) from 2013 to 2023. Data sources: Statistics Portugal (INE, 2024) for Portugal, and Ministerio de Agricultura, Pesca y Alimentación (MAPA, 2024) for Spain. *Significant at ρ <0.05.

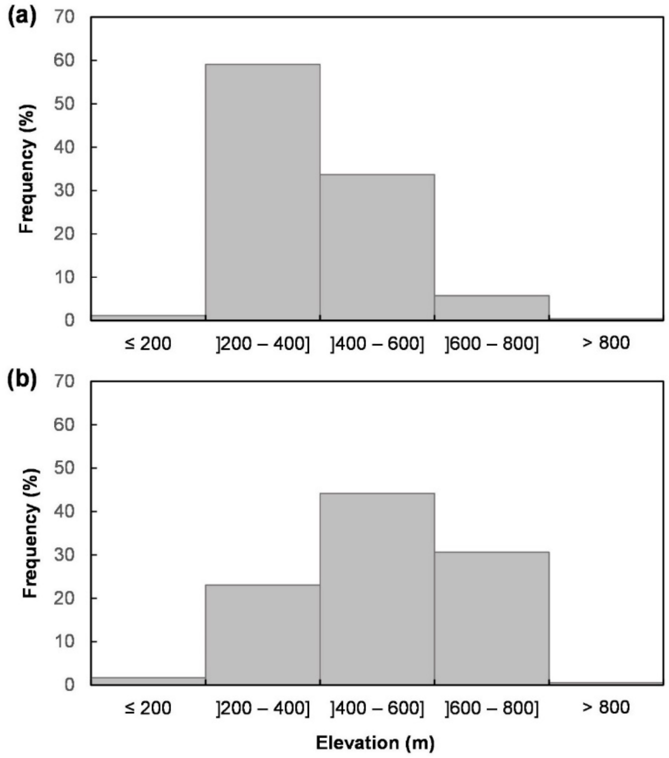

Figure S2. Histogram of elevation in both regions: (a) Badajoz (BA) province; (b) "Trás-os-Montes" (TM) agrarian region.

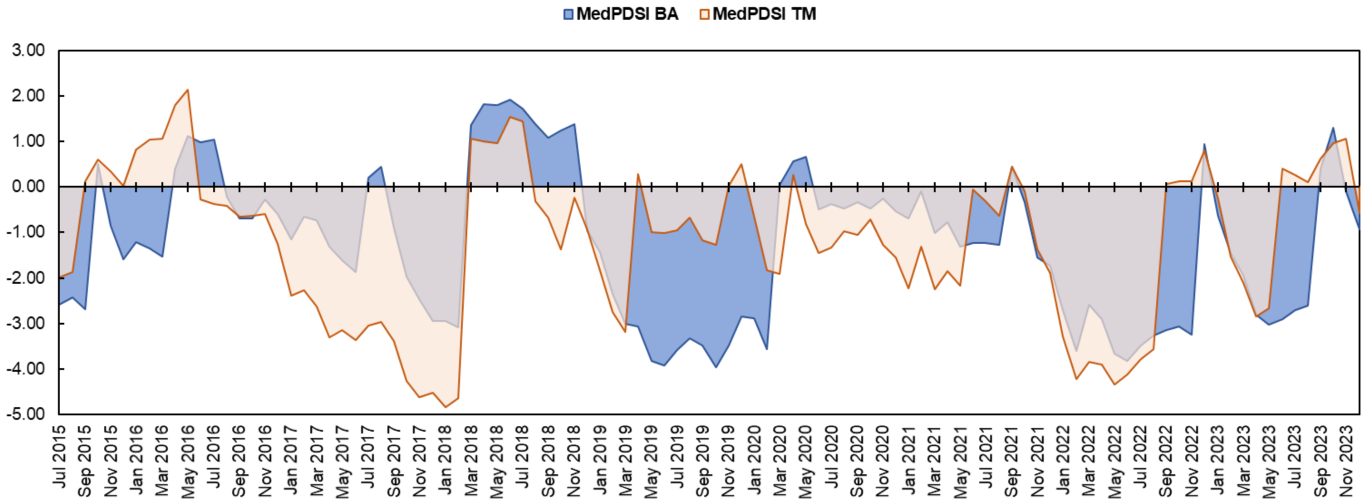

Figure S3. Comparison of MedPDSI between Badajoz (BA) province and "Trás-os-Montes" (TM) agrarian region.

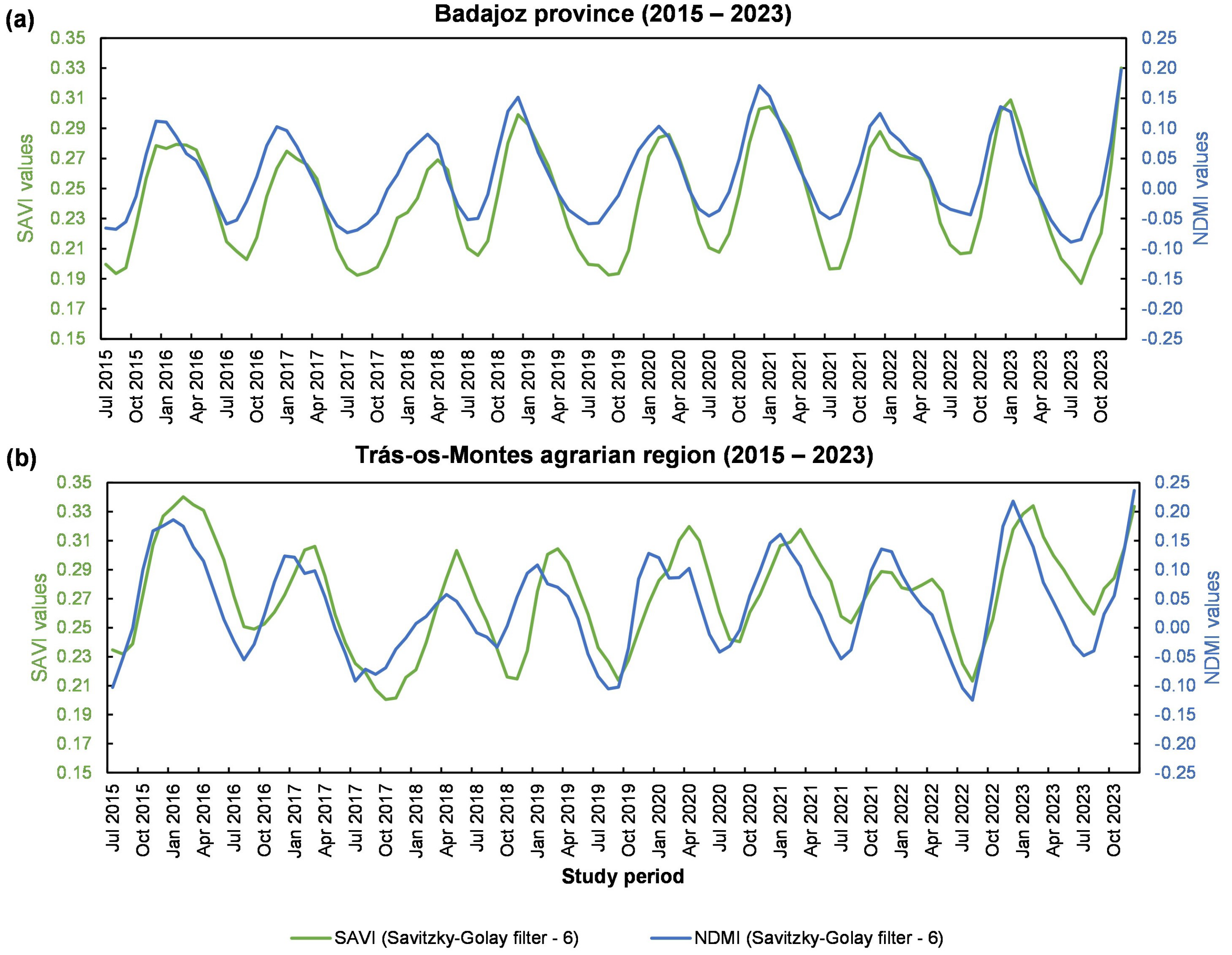

Figure S4. Comparison of SAVI and NDMI indices: (a) Badajoz (BA) province, (b) “Trás-os-Montes” (TM) agrarian region.
